# In the Loop: The Organization of Team-Based Communication in a Patient-Centered Clinical Collaboration System

**DOI:** 10.2196/humanfactors.4996

**Published:** 2016-03-24

**Authors:** Allison M Kurahashi, Peter B Weinstein, Trevor Jamieson, Jennifer N Stinson, Joseph A Cafazzo, Bhadra Lokuge, Plinio P Morita, Eyal Cohen, Adam Rapoport, Andrea Bezjak, Amna Husain

**Affiliations:** ^1^ Temmy Latner Centre for Palliative Care Mount Sinai Hospital Toronto, ON Canada; ^2^ Healthcare Human Factors Centre for Global eHealth Innovation, Techna Institute University Health Network Toronto, ON Canada; ^3^ Division of General Internal Medicine St. Michael's Hospital Toronto, ON Canada; ^4^ Institute for Health Systems Solutions and Virtual Care Women's College Hospital Toronto, ON Canada; ^5^ Department of Medicine University of Toronto Toronto, ON Canada; ^6^ Child Health Evaluative Sciences The Hospital for Sick Children Toronto, ON Canada; ^7^ Lawrence S Bloomberg Faculty of Nursing University of Toronto Toronto, ON Canada; ^8^ Institute of Health Policy, Management and Evaluation University of Toronto Toronto, ON Canada; ^9^ Institute of Biomaterials and Biomedical Engineering University of Toronto Toronto, ON Canada; ^10^ Commonwealth Fund/Canadian Foundation for Healthcare Improvement Commonwealth Fund New York, NY United States; ^11^ Department of Paediatrics University of Toronto Toronto, ON Canada; ^12^ Paediatric Advanced Care Team The Hospital for Sick Children Toronto, ON Canada; ^13^ Emily's House Children's Hospice Toronto, ON Canada; ^14^ Department of Family and Community Medicine University of Toronto Toronto, ON Canada; ^15^ Department of Radiation Oncology Princess Margaret Cancer Centre University of Toronto Toronto, ON Canada

**Keywords:** collaborative care, patient-centered care, patient engagement, chronic disease, communication, Internet communication tools, Internet communication technologies

## Abstract

**Background:**

We describe the development and evaluation of a secure Web-based system for the purpose of collaborative care called Loop. Loop assembles the team of care with the patient as an integral member of the team in a secure space.

**Objective:**

The objectives of this paper are to present the iterative design of the separate views for health care providers (HCPs) within each patient’s secure space and examine patients’, caregivers’, and HCPs’ perspectives on this separate view for HCP-only communication.

**Methods:**

The overall research program includes cycles of ethnography, prototyping, usability testing, and pilot testing. This paper describes the usability testing phase that directly informed development. A descriptive qualitative approach was used to analyze participant perspectives that emerged during usability testing.

**Results:**

During usability testing, we sampled 89 participants from three user groups: 23 patients, 19 caregivers, and 47 HCPs. Almost all perspectives from the three user groups supported the need for an HCP-only communication view. In an earlier prototype, the visual presentation caused confusion among HCPs when reading and composing messages about whether a message was visible to the patient. Usability testing guided us to design a more deliberate distinction between posting in the Patient and Team view and the Health Care Provider Only view at the time of composing a message, which once posted is distinguished by an icon.

**Conclusions:**

The team made a decision to incorporate an HCP-only communication view based on findings during earlier phases of work. During usability testing we tested the separate communication views, and all groups supported this partition. We spent considerable effort designing the partition; however, preliminary findings from the next phase of evaluation, pilot testing, show that the Patient and Team communication is predominantly being used. This demonstrates the importance of a subsequent phase of the clinical trial of Loop to validate the concept and design.

## Introduction

### Overview

As the complexity of health care increases, we are recognizing the limits of current models of program-centered and specialty-centered care [1-3]. Patient-centered care and patient engagement have the potential to substantially improve outcomes in the health care system [4-6]. The penetration of Internet and mobile technologies makes it possible to envision new systems for interactive communication that follow the patient across the continuum of care. In this paper, we present aspects of the design, development, and evaluation of such a system. The system, called Loop, uses social networking principles to assemble the patient’s actual team of care and include the patient as an integral member of the team for the purpose of collaborative care.

### The Gap

In the United States, 84% of all health care spending in 2006 was for the 50% of the population who have one or more chronic medical conditions [7,8]. In Canada, chronic disease contributes disproportionately to the total economic cost of illness [9,10]. Globally, chronic disease is predicted to increase both in prevalence and complexity. “The most common chronic condition experienced by adults is multimorbidity, the coexistence of multiple chronic diseases or conditions” [11]. These are patients with complex chronic disease who require multiple health providers and have unique needs, disabilities, or functional limitations [12]. Currently, health care is organized in organizational and disease-specific silos that the patient moves across frequently and unpredictably, eliciting a broad call for transformative solutions [13,14]. Wagner’s chronic care model [15-17], endorsed in several countries including the United States and Canada, proposes a roadmap for effective management that calls for “planned, proactive seamless care in which the clients are full participants in managing their care and are supported to do this at all points by the system” [18]. However, there are few systems to enable engagement and collaboration. Understanding the gap and potential solution grew from our team’s experience, which spans diverse populations with chronic and complex care needs including home palliative care, cancer care, acute to ambulatory care transitions, adolescents and young adults with cancer (AYAC), and children with medical complexity (CMC). Lack of communication is a problem identified across all these populations; fostering communication is a key process if we are to achieve continuity of care and comanagement [19,20]—a goal endorsed by all stakeholders [21,22]. Comanagement, or collaborative care, requires more than a passive sharing of electronic health records (EHRs). It requires ongoing, interactive, and contextual communication among team members [1]. A report from the American Medical Informatics Association’s 2013 Policy Meeting on patient-centered care highlights this: “EHRs are necessary but not sufficient to engage patients and foster improvements in the quality of care . . . health information needs to flow across the health care continuum” [6].

### The Solution: Loop

The evidence supports collaborative care as the keystone to chronic disease management, the patient and caregiver as integral partners in care, and communication as central to achieving these objectives [4,13,15]. We propose a solution using emerging social networking technologies: a Web-based clinical collaboration system for complex chronic disease patients. In Loop, each team of care centered on a patient, or Patient Loop, consists of the patient, the caregiver, and the health care providers (HCPs) involved in the patient’s care. Each Patient Loop is a secure space partitioned from every other Patient Loop, and those with access must be involved in the patient’s care and authenticated to join. While users are not provided with specific instructions, Loop is designed to encourage them to communicate questions, updates, and clarifications about care plans. Loop allows team members, including the patient, to indicate their preferences and check their understanding of the care plan. The communication is visible to the team but specific members of the team may be tagged. Therefore, in terms of types of communication there could be an exchange between patient (or caregiver) and HCP or between HCPs in the team. The purpose of Loop is for team members to arrive at care plans together and work toward a shared set of goals.

### Rationale for a Web-Based Clinical Collaboration System

Several studies report the desirability, acceptability, and manageability of messaging systems focused on patient-physician communication [23-25]. Two studies evaluating patient-HCP messaging systems did not find detectable differences in the volume of communication with these systems [23,24]. Results show participants had increased satisfaction with communication, improvement in workflow, and overall positive attitudes towards online communication [23,24,26].

We conducted a search for existing systems primarily in the United States and Canada and approached vendors and groups working in the communication space [27-30]. In the context of large health care organizations in the United States, communication is embedded within each organization’s information technology. However, this does not work outside the confines of a large health network in the United States or in the contexts of other countries. For example in Canada, patients move across the single-payer system without the restrictions imposed by insurance providers or organizations. We studied a number of messaging tools developed for use within the confines of a hospital in a large urban center in Canada [2]. Separate development has led to multiple tools for similar functions with no reach beyond the organization. HCPs reported that tools are not integrated into their work flow, resulting in decreased efficiency and tools being used in incorrect ways [31]. We embarked on the research and development of Loop when communication tools for direct patient care were just emerging. Our literature review revealed a few communication tools with potential to extend beyond organizational boundaries; however, they are limited in a number of ways [27-30]. A patient-held record (PHR) may have potential to be used as a communication tool, but existing PHRs are institutionally sponsored, limited to the institution’s patients, and do not have the functionality of providing a space for HCPs to communicate for collaborative care [32,33]. EHRs focus on transmitting medical reports as a means of communication without enabling interactive exchange. Still other tools limit communication to certain groups (eg, HCPs only) or to certain forms (eg, private one-on-one messaging) [26]. None of the tools had the integrated functionality we envisioned: a focus on communication for direct patient care in the community, a networking structure, and separate but integrated communication spaces for patients and HCPs. Recently, a handful of tools that overlap some of the functions of Loop have emerged [34-37]. Our program of research on Loop, with its iterative user-driven development and its robust evaluation, contributes important learning to this emerging field of eHealth tools for care coordination.

Existing systems continue to be organization-centric and propagate a model that is minimally collaborative, excludes key players, and is ill-suited to the complexity of health care [31,38]. Loop is interactive, asynchronous communication that enables the patient's team of care to be assembled, no matter what their profession, where they practice, or what their organizational affiliation is; and it includes patients, caregivers, and health care professionals in the communication. The Web-based platform allows Loop to reach beyond organizational boundaries. In future phases, we will use plug-in or application programming interface technology to link to the different EMRs across organizations. We envision that Loop will serve as a communication layer linked to other eHealth tools in a personalized dashboard.

### The Development of Loop

**Figure 1:**
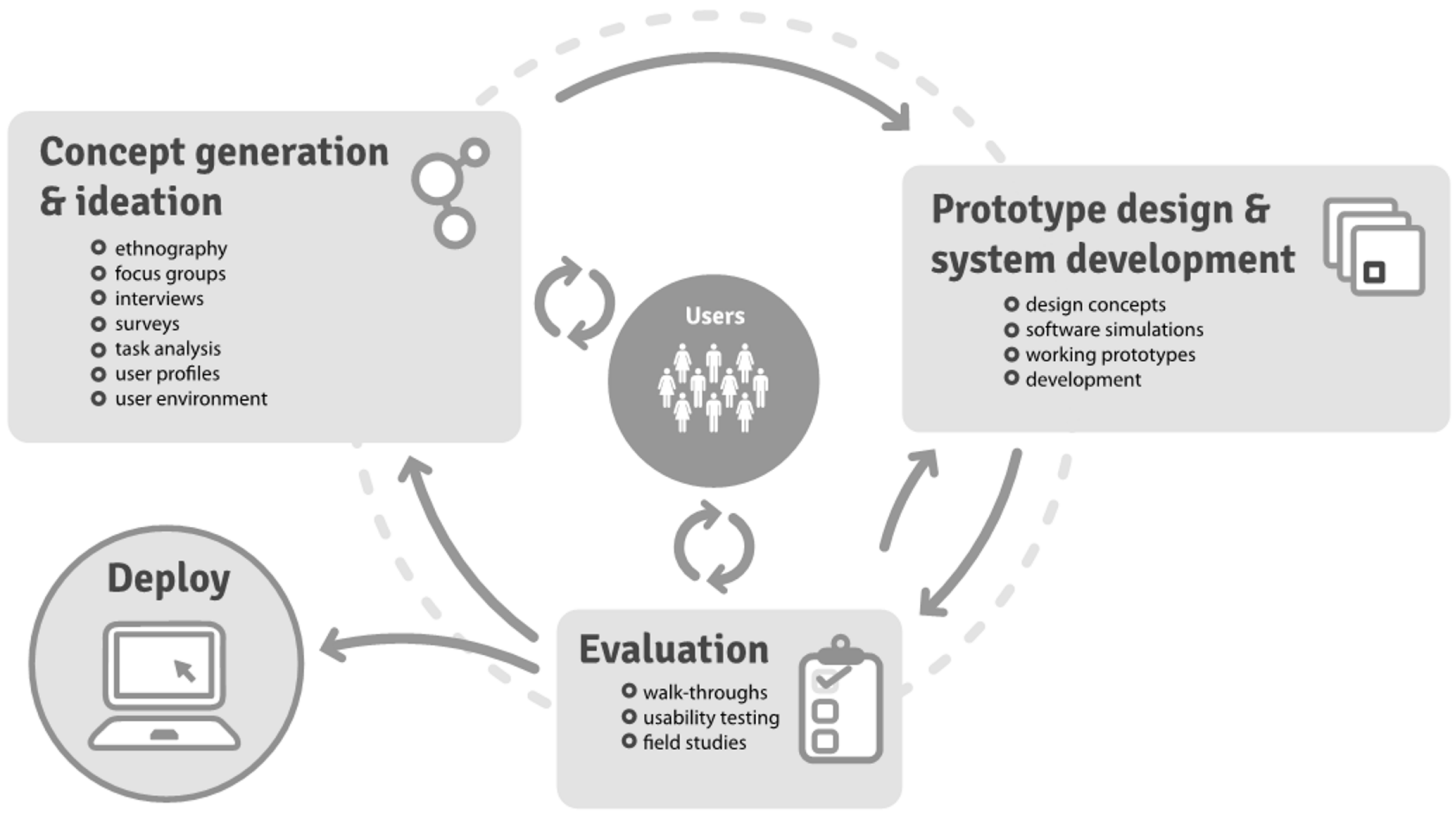
User-centered design process extracted from McCurdie et al [27].

In line with existing recommendations to rigorously evaluate eHealth systems throughout all stages of their life cycles [24], we chose a sequential plan of research following the Medical Research Council framework for complex interventions [39]. We embedded an iterative stakeholder engagement process based on user-centered design (UCD) [40,41] and participatory design methods (Figure 1). User or end-user refers to patients, caregivers, and HCPs who would use Loop in planning or coordinating care. Participatory design calls for the engagement of clinicians, researchers, developers, designers, end-users, and the technology itself throughout development [42]. Thus research and development have been integrally linked, and the various research activities have been continuous and reflexive. Through this research spanning more than 5 years, we have developed and tested the Loop prototype in simulated and real settings.

The concept of open versus private communication within the team has been at the core of the development of Loop. At inception, the research team had an idea that open communication between the members of the team of care, regardless of what their role or where they practiced, would be transformative. Previous literature has indicated that having clinical discussions in the presence of patients and families during bedside rounds improves communication and transparency [43,44]. Despite these benefits, the authors report parent and HCP concern about negative emotional responses and confusion that may result from technical discussion [43,44] and the need for “pre-rounding” or “re-rounding” away from patients and families to have uninhibited conversations [44]. Prior to the usability testing phase that is the focus of this paper, all user groups endorsed two separate communication spaces within a patient’s secure space: one for the entire team including patients and caregivers and another for HCPs only. We carried this knowledge forward when creating prototypes by including an option for HCP-only communication. The perspectives on the two separate views that emerged in usability testing are the focus of this paper.

### Objectives

The objectives of this paper are to present the iterative design of the separate view for HCPs within each patient’s secure space and examine patient, caregiver, and HCP perspectives on this separate view. While providing feedback about the visual design of the separate view during usability testing, participants also shared perspectives about HCPs communicating with each other without patients or caregivers able to view the communication within Loop. The focus of the analysis presented in this paper is derived solely from the usability testing, and we limit our description to the usability testing phase.

## Methods

### Summary of Phases of Work

Our search for existing systems found none with team-based communication that included both the patient and the team of HCPs, was cross-organizational, and followed the patient across the entire health system. We used UCD methods (Figure 1) to engage the final users of the product as active participants in the design process and gather user needs as product requirements [41]. Specifically, we employed the following components of UCD: (1) ethnography [45], (2) affinity diagramming [46], (3) cooperative prototyping [47], (4) dramatic simulation activities [47], (5) usability testing and prototyping [48], and (6) pilot testing. This paper focuses on usability testing while briefly discussing the other activities for context.

Usability testing followed a descriptive qualitative method [49] and content analysis, which aims to summarize the informational content of verbal and visual data [50,51]. This analysis was reflexive and interactive throughout usability testing.

### Population

We recruited a convenience sample of participants from the following populations: adult cancer, adolescents and young adults with cancer (AYAC), and children with medical complexity (CMC). We recruited patients with cancer, caregivers of patients with cancer, and HCPs representing a variety of disciplines involved in cancer care. In the CMC area, we recruited parents of CMC patients and HCPs involved in their care. We obtained relevant institutional review board approvals and informed consent from all participants.

### Usability Testing and Prototyping

#### Usability Testing Procedure

During three rounds of usability testing in simulation labs, participants were asked to follow the think-aloud protocol while interacting with prototypes of progressing fidelity [48]. The prototypes were prepopulated with messages based on realistic patient stories and served as the foundation for participants to interact with and respond to. A facilitator provided task-oriented scenarios guiding participant interactions with the system and asked questions about participant experiences. Data were collected using screen and audio capture and by note-takers in an adjacent observation room. In addition, we tested the prototype offsite following the same simulation protocol with a different sample of patients and caregivers in their homes and HCPs in their practice settings. Offsite usability testing occurred concurrent with and in between rounds of simulation testing in labs. In all instances, participants were asked to complete a pretest survey for information on demographics and comfort with technology. Each session of usability testing involved a unique participant with the exception of one caregiver who participated in two usability testing sessions but is counted as one participant. All interviews were transcribed verbatim.

#### Applying Usability Testing Feedback to Prototype Development

**Figure 2:**
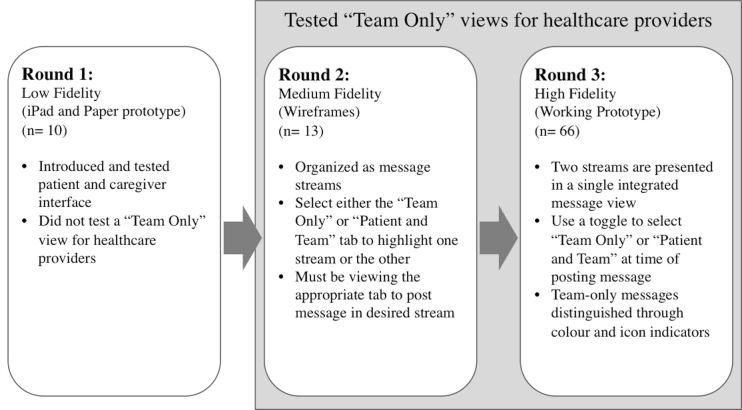
Usability testing and prototype progression.

A basic interactive prototype was created using Axure RP version 6.5. Prototyping early gave participants something to respond to when providing feedback about major design principles and required features. This set of specifications using screenshots and detailed descriptions informed the design and development of low-, medium-, and high-fidelity prototypes within cycles of usability testing as described above. A low-fidelity prototype focused on the introduction of a patient and caregiver interface and was a necessary step in the evolution to the later prototypes. The low-fidelity prototype did not evaluate separate streams in the HCP view. The interactive medium- and high-fidelity prototypes were the first instances where the usability and acceptability of HCP-only messaging could be tested (Figure 2).

### Qualitative Analysis of Usability Testing

The content of usability testing interviews was presented and discussed in weekly project team meetings. Consensus was achieved on design principles that were tracked in a user specification document that served as an audit trail for the process. Additionally, emerging concepts and major decisions of the team were captured in meeting notes. Interview transcripts were independently coded in NVivo version 10 (QSR International) by three reviewers, who met initially to arrive at consensus for a coding framework and continually to discuss subsequent coding application. At key points, two senior team members (AH and JS) reviewed the framework. The ongoing process of review has given the team a grounding in the data to inform further in-depth analysis focused on emerging concepts. Usability testing interview data were reviewed and selectively coded to identify participant perceptions about private messaging among HCPs and open communication with all team members including the patient and caregiver. Quotes were extracted from references coded in the preliminary categories of *visibility of messages*, *team composition*, *composing a message*, and *medical terminology*. As themes emerged, queries were run with the keywords *private*, *conversation*, *confusion*, and *anxiety* to identify any additional quotes related to open and closed communication. Through this process, two reviewers refined the initial categories into emergent themes.

## Results

### Population

Across all the activities, we had a convenience sample of 150 participants from the CMC, AYAC, and adult cancer populations (tables 1-4). A subset composed of 89 participants took part in usability testing. In this subset, there were 23 patients, 19 caregivers, and 47 HCPs. Results of the usability testing and its impact on development of the prototype are described together because one activity continually informed the other. With regard to access to technology, Internet penetration at home ranged from 91% to 100% across populations and user groups in this sample. Although the numbers are small in each user subgroup, the findings suggest trends: HCPs had the most use of computers and access to Internet both at work and home, and AYAC patients had the most comfort with smartphones and social media.

**Table 1:** Number of participants involved across all activities.

		Role		
Data collection activity	Population	Health care provider	Patient/ caregiver	Activity total
**Focus groups, interviews, and ethnography**				
	Adult cancer	14	9	
	AYAC	7	0	
	CMC	5	0	
	Total	26	9	35
**Usability testing** ^a^				
	Adult cancer	19	20	
	AYAC	16	15	
	CMC	12	7	
	Total	47	42	89
**Pilot testing**				
	Adult cancer	6	3	
	AYAC	6	3	
	CMC	6	2	
	Total	18	8	26
Total		91	59	150

^a^Findings of the usability testing are the focus of this paper.

**Table 2:** Patient participant profile data (usability testing only).

		Adult cancer N=8	CMC N=0	AYAC N=15
Female, n (%)		5 (62)	—	5 (33)
Age, years, median (range)		61 (40-79)	—	17 (15-26)
**Diagnosis, n (%)**				
	Breast cancer	0 (0)	—	—
	Colorectal cancer	0 (0)	—	—
	Lung cancer	2 (25)	—	—
	Ovarian cancer	1 (12)	—	—
	ALL	—	—	3 (20)
	AML	—	—	2 (13)
	Ewing sarcoma	—	—	1 (7)
	Rhabdomyosarcoma	—	—	1 (7)
	Non-Hodgkin lymphoma	—	—	1 (7)
	Osteosarcoma	—	—	2 (13)
	Other	5 (62)	—	5 (33)
Use a computer at work/school, n (%)		5 (63)	—	13 (87)
Use a computer at home, n (%)		7 (88)	—	14 (93)
Use the Internet at home, n (%)		7 (88)	—	14 (93)
**Comfortable using, n (%)**			—	
	Computer	5 (63)	—	15 (100)
	Smartphone	3 (38)	—	15 (100)
	Internet	6 (75)	—	15 (100)
	Email	7 (88)	—	14 (93)
	Instant messaging	4 (50)	—	15 (100)
	Social media	2 (25)	—	13 (87)
**Hours spent on computer per day**				
	<1	2 (25)	—	0 (0)
	1-7	5 (63)	—	12 (80)
	>7	1 (13)	—	3 (20)
**Hours spent on Internet per day**				
	<1	2 (25)	—	1 (7)
	1-7	6 (75)	—	12 (80)
	>7	0 (0)	—	2 (13)

**Table 3:** Caregiver participant profile data (usability testing only).

		Adult cancer N=12	CMC N=7	AYAC N=0
Female, n (%)		7 (58)	6 (86)	—
Age, years, median (range)		56.5 (31-72)	37 (32-45)	—
**Caregiver type, n (%)**				
	Spouse	4 (33)	0 (0)	—
	Son/daughter	5 (42)	0 (0)	—
	Mother/father	1 (8)	7 (100)	—
	Other	2 (17)	0 (0)	—
Use a computer at work/school, n (%)		10 (91)	6 (86)	—
Use a computer at home, n (%)		11 (92)	7 (100)	—
Use the Internet at home, n (%)		11 (92)	6 (100)	—
**Comfortable using, n (%)**				—
	Computer	11 (92)	7 (100)	—
	Smartphone	10 (83)	5 (71)	—
	Internet	11 (92)	7 (100)	—
	Email	11 (92)	7 (100)	—
	Instant messaging	11 (92)	7 (100)	—
	Social media	6 (50)	4 (57)	—
**Hours spent on computer per day**				—
	<1	1 (8)	0 (0)	—
	1-7	8 (67)	3 (44)	—
	>7	3 (25)	4 (57)	—
**Hours spent on Internet per day**				—
	<1	1 (8)	0 (0)	—
	1-7	10 (83)	6 (86)	—
	>7	1 (8)	1 (14)	—

**Table 4:** Health care provider participant profile data (usability testing only).

		Adult cancer N=19^a^	CMC N=11^a^	AYAC N=16^a^
Female, n (%)		13 (68)	10 (91)	14 (87)
**Age, n (%)**				
	20-29	0 (0)	1 (9)	1 (6)
	30-39	6 (32)	2 (18)	5 (31)
	40-49	6 (32)	4 (36)	7 (44)
	50-59	3 (16)	3 (27)	2 (12)
	60-69	4 (21)	1 (9)	1 (6)
Years in health care, median (range)		15 (3-40)	20 (3-35)	18.5 (2.5-39)
**Profession, n (%)**				
	Family physician	7 (37)	0 (0)	—
	Community nurse	0 (0)	2 (18)	—
	Palliative care physician specialist	2 (10)	0 (0)	—
	Medical oncologist	1 (5)	0 (0)	—
	Other specialist	6 (32)	1 (9)	—
	Case manager	1 (5)	3 (27)	—
	Other	2 (10)	3 (27)	—
	General pediatrician	0 (0)	2 (18)	—
	Physician	—	—	5 (31)
	Advanced practice nurse	—	—	8 (50)
	Nurse	—	—	2 (12)
	Psychologist	—	—	1 (6)
Use a computer at work/school, n (%)		19 (100)	11 (100)	—
Use a computer at home, n (%)		19 (100)	11 (100)	—
Use the Internet at home, n (%)		19 (100)	11 (100)	—
**Comfortable using, n (%)**				
	Computer	19 (100)	11 (100)	—
	Smartphone	18 (95)	9 (82)	—
	Internet	19 (100)	11 (100)	—
	Email	18 (100)	10 (100)	—
	Instant messaging	17 (94)	10 (100)	—
	Social media	8 (44)	5 (50)	—
**Hours spent on computer per day**				
	<1	0 (0)	0 (0)	—
	1-7	13 (68)	6 (55)	—
	>7	6 (32)	5 (46)	—
**Hours spent on Internet per day**				
	<1	0 (0)	1 (9)	—
	1-7	14 (74)	7 (64)	—
	>7	5 (26)	3 (27)	—

^a^Percentages are calculated based on the number of answers submitted. Not all questions were completed by all participants.

### Objective 1: Usability Testing and Prototyping

**Figure 3:**
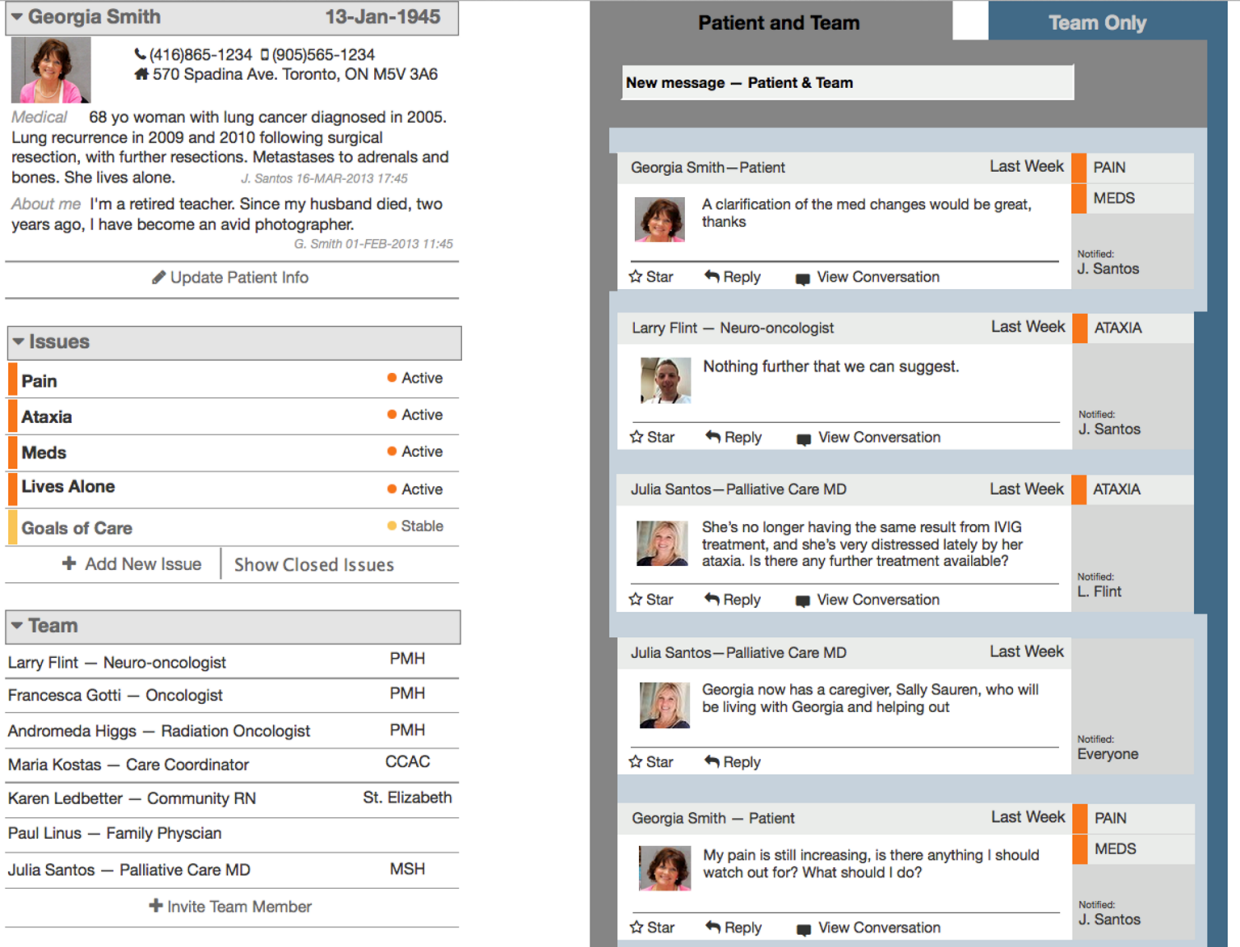
Medium-fidelity prototype of health care provider view with two message streams distinguished by visual treatment. Scenario and mock-up based on actual patient case.

The medium-fidelity prototype was the first iteration to have interfaces for patients and caregivers in addition to the interface for HCPs (Figure 2). In the prototype (Figure 3), the HCP view was organized as two streams of messages: one with messages visible to the patient and the other to HCPs only. Each stream was given a different visual treatment, and HCPs were able to select the stream of conversation to join from this view. The patient and caregiver view had only one stream of messages. This organization caused confusion for some HCPs, who found it hard to tell whether the patient was involved in a conversation. Therefore, this design did not meet our objective of an intuitive user experience. Further analysis indicated that for HCPs the distinction was more important while composing and sending messages than while viewing messages.

**Figure 4:**
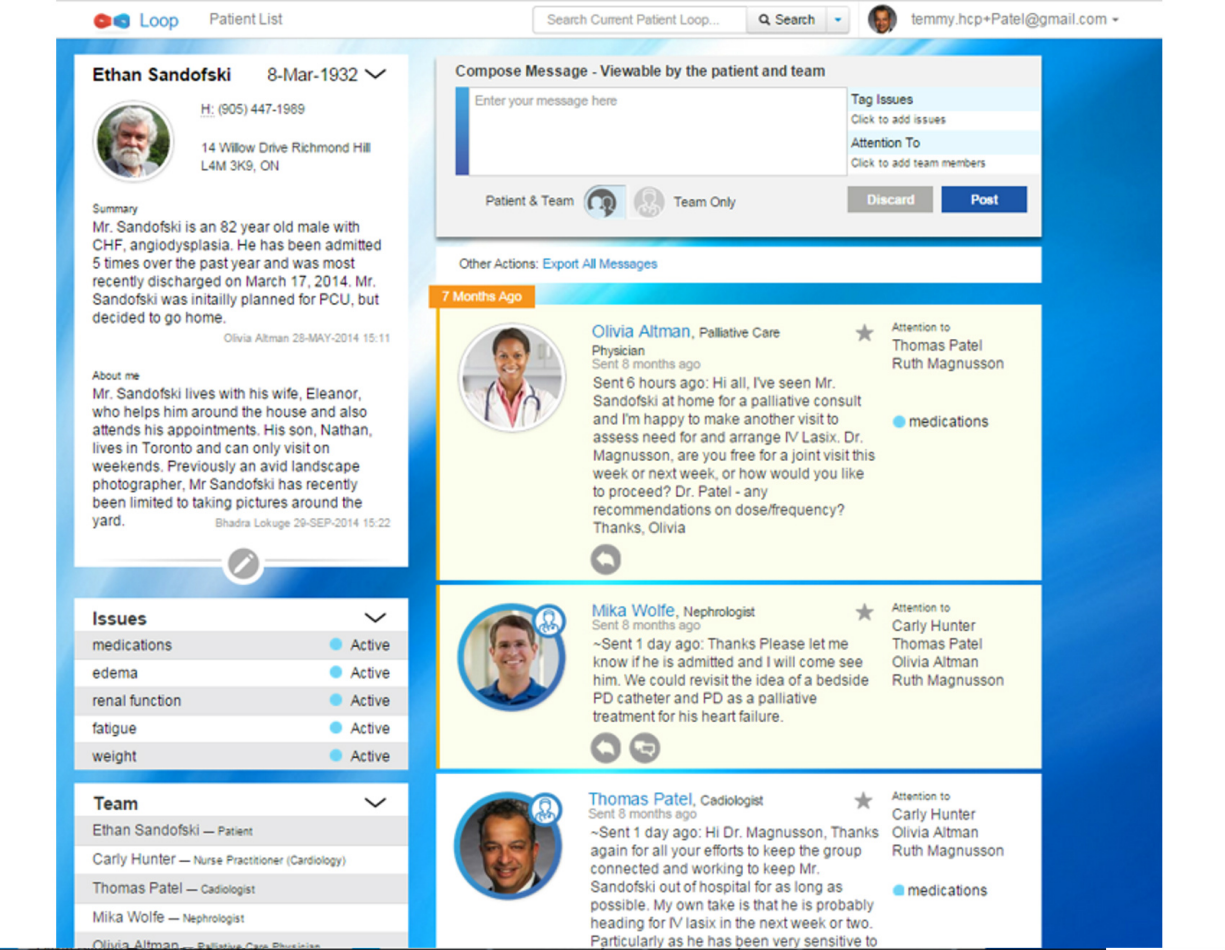
High-fidelity prototype version of Loop with Patient and Team and Team Only toggle from message compose box.

In the high-fidelity prototype (Figure 4), we incorporated this learning by removing the visual treatment of the two streams and introducing a prominent toggle (*Patient and Team* and *Team Only*) in the compose message box prompting HCPs to make a selection at the time of posting the message. Once posted, any message for HCPs only is distinguished by an icon. The reply message is by default an HCP Team Only message unless Patient and Team is actively selected in the toggle.

### Objective 2: Qualitative Analysis of Usability Testing

Analysis of usability testing transcripts found that the concept of open messaging between the team and patient and caregiver was new to participants across all user groups. In both the medium- and high-fidelity prototype cycles of usability testing, the vast majority expressed the need for a separate space for HCP communication within the Patient Loop, with only a few participants concerned that this would disenfranchise patients and caregivers.

Those who expressed a need for a separate space for HCP communication had two main reasons. First, HCPs may communicate more freely and efficiently if patients are not part of the conversation.

In a Team Only circumstance, you can probably say things in a little bit more free-form or with less restraint. And that’s partly because you need to be that frank. You need to say listen, this is very worrisome, don’t know what to say to mom, let’s have a conversation about this and this is where that Team Only option is good.

HCP #1, CMC

Theoretically, I buy into the idea that the patient should be part of everything. But it really does change what you’re able to put in the message. So, in the real world, when you’re really in a hurry, and you really want to get some stuff out there, you might not be able to.

HCP #2, adult cancer

And we’re not necessarily accustomed to talking to other health professionals with the patient aware of every word that’s said or the way it’s said or the way it could easily be misinterpreted . . . those kinds of things.

HCP #3, adult cancer

Second, messages could contain information that causes confusion or anxiety for patients and caregivers. For example, patients and caregivers may experience confusion after viewing a message about a preliminary stage of planning.

If it’s something that is in very preliminary discussion and it’s not a possibility but it’s a thought, I probably would not wanna be privy to that.

Patient #1, AYAC

. . it would be beneficial for the doctors to talk amongst themselves before they give you an answer that might mislead you to [think] something else . . .

Patient #2, AYAC

You don’t necessarily want to hear everything that the doctors are discussing . . . You want to hear the end discussion, you don’t want to be more confused.

Caregiver #1, CMC

In circumstances where you want to have an internal conversation about consultation and you’re not really sure what the plan is and it’s not necessarily for the family to know because the family typically wants to know what the plan is. They don’t necessarily want to be part of the particulars around the plan . . .

HCP #1, CMC

In addition, patients and caregivers may experience anxiety if messages contain new information about their disease or treatment.

Well, this [the message stream in Loop] is very detailed about what happened and how it went and I think if a patient is willing to read all this, is comfortable reading that, it is good. But what if a patient is not comfortable reading whatever details there are and how bad it is?

Caregiver #2, adult cancer

. . . It’s not saying that the patient is not going to get it, but there’s a way to disclose that information where the patient could have some family there and instead of doing it this way, where they might just get it as an email. It’s so much more impersonal than actually sitting down and having a conversation.

HCP #4, AYAC

So, if it’s only a medical [term] that she’s unlikely to understand, or might get freaked out about.

HCP #5, adult cancer

. . . there are some times where you’re in a formative stage and it’s not probably to the patient’s interest to talk about really what’s his prognosis and have we determined that, before I say it to him.

HCP #6, adult cancer

These perspectives supporting a separate communication space for HCPs based on protection of the patient and efficiency for the HCP should be considered in light of the few divergent perspectives favoring all communication be open to patients and caregivers.

I understand both sides being health care people want to talk about health care issues. And if you’re talking about how to disclose a diagnosis to a patient, you can’t have the patient reading that. At the same time, I think that [separate views] makes this a health care provider–favored tool rather than a patient-favored tool.

HCP #7, CMC

. . so the only thing I’m questioning with this is whether it should go to the patient, but . . . I’m assuming the point of it all is, that’s why I’m just trying to . . . I was just sort of struggling with how to phrase it. [Pause] . . . because it’s such a sensitive issue but then . . . I mean I think it should because it’s obviously a team issue that the patient’s brought forward. And I assume that part of this is to be completely transparent. Is that to have all discussions and that for the patient to know . . . what team members are saying . . . that there’s a transparency to it.

HCP #8, AYAC

Patients, caregivers, and HCPs all believe that there are different considerations, conventions, and language governing conversations between HCPs versus those between HCPs and patients. The challenge for Loop is how to best weave these conversations in one platform, respecting the prevailing perspectives but keeping true to the aim of changing the status quo as it relates to patient engagement.

## Discussion

### Principal Findings

This paper describes the perspectives of patients, caregivers, and HCPs on an HCP-only communication space within a patient’s secure communication space. All user groups, including patients and caregivers, support HCP-only communication. Usability testing informed the design of this partition in successive prototypes. The overall program of research has explored this core concept as well. At inception, the team had an idea that open communication, where all messages were visible to the patient and the entire professional team, would democratize communication and mitigate the hierarchies that exist in health care. Consistent with prior ethnographic work, usability testing showed that almost all end-users including patients and caregivers endorsed a separate HCP message view. The challenge in Loop is that it must serve the communication needs of all its user groups: patients, caregivers, and HCPs. Loop must be able to accommodate the different considerations that govern communication between HCPs versus communication between patients and HCPs. At the same time, it must provide the flexibility to engage patients in their plan of care. We found similar perspectives reflected in the literature. While patients want to be engaged in health care decisions, they trust their clinicians to have the knowledge and skills to arrive at and propose appropriate options for care [52]. Patients want to be engaged in the decision-making process according to their preferences for receiving communication [53]. In Loop, this preference can be accommodated within each team. Each Patient Loop is expected to be a self-regulating microenvironment based on the characteristics, context, and behavior of team members. It is expected that team agreements and roles will change and evolve over time through interactions in Loop.

Although the data from pilot testing in real-world teams will be the focus of a subsequent paper, preliminary results suggest that the majority of messages exchanged are between patients and HCPs and therefore are in the open Patient and Team view. This illustrates the need for phases of evaluation of implementation and effectiveness that we are currently conducting. Our sequential approach to evaluation is supported by two reviews of health information technology used to facilitate communication; both call for evaluation that uses methodological standards such as the Medical Research Council framework [54,55]. Additionally, a 2015 scoping review of information and communication technology (ICT) supports our UCD approach [54]; only 6% of 350 studies identified for inclusion evaluated usability of the tool to any degree. The authors of the review point out the need for usability testing: “*This is disturbing since usability is an important factor for the acceptability of ICT by its users, and the lack of attention paid to usability in the reviewed studies indicates that there would be much to be gained from this*” [54].

### Limitations

One limitation of expert and user feedback is that end-users may not know what they need until using a system in practice [56]. Additionally, end-users often have wish lists that are specific to their context, which make contradictory demands on the system and make it less usable for other end-users [57]. Therefore, we analyzed and prioritized the feedback in a group with clinical, research, development, and design representation. Usability testing in a lab or simulated setting does not allow for evaluation of how a system would be used in a real-world setting and how it fits into workflow. This will be tested in subsequent phases of our research.

The sample may be biased toward individuals who were more engaged, favorably disposed to technology, and functionally capable. We will need to address the challenge of accessibility, adoption, and scaling in the next phase of the work.

### Comparison With Prior Work

We found no studies examining the issue of separate message views for patients and HCPs in a team-based communication system. In addition to the studies on patient-physician communication referenced previously [52,53], there is a body of literature that examines the perceived benefits and concerns associated with bedside rounds conducted in the presence of the patient and caregiver [58]. Grzyb et al [43] surveyed parents of children admitted to the neonatal intensive care unit and medical trainees who rotated through the unit to solicit views on parents being in attendance at rounds. Stickney et al [44] interviewed parents of children admitted to an intensive care unit and HCPs (nurse, residents, fellows, and attending intensive care unit physicians) about parents’ and providers’ goals and expectations for participation in morning rounds. Our findings echo the findings in these papers: HCPs did not like discussing unfavorable prognoses in front of parents and felt that discussion among providers was inhibited. They worried about information being misinterpreted and a “negative emotional response to unwelcome news” and felt that parents attending rounds made for longer rounds [44]. Parents had polarized views on whether they should be given bad news during rounds, felt it might be upsetting to hear health care providers express uncertainty about their child’s condition or treatment [43], and were concerned about being confused by the technical nature of the discussion [44]. Parents felt more included in their child’s care when they were present for bedside rounds [44].

There are only a handful of systems like Loop (ie, tools for cross-sectoral collaboration with team-based communication as their focus) in use today. The application of networking technologies to communication in health care is an emerging field. As Bates states in a recent article: “*If organizations want to succeed in improving quality and reducing costs, providing better care coordination is one of the most important keys. However, the electronic health records of today do not yet truly enable care coordination. Even the leading US organizations in care coordination do not yet have robust electronic tools for doing this—making this a key frontier for clinical informatics”* [38].

Any intervention developed for the purpose of clinical communication about the patient and involving the patient must be patient-centered. The 2015 scoping review of ICT states that “*hardly any of the interventions could be regarded as ‘fully’ person-centered care (PCC) meeting the 3 routines of initiating the partnership (patient narratives), working the partnership (shared decision making), and safeguarding the partnership (documenting the narrative)*” [54]. Loop facilitates each of these processes. Its authors further state that “*shared decision making, personal information sharing, and setting up a care plan enabled by ICT seem to be relatively new*” [54].

In describing a system like Loop, it is important to address the question of feasibility. We acknowledge that the problem of poor communication is not just a technology problem. Implementing Loop requires considering the characteristics of individuals, organizations, incentives, and policies. At the individual level, HCPs fear a tsunami of electronic messages or an erosion of the rules that have traditionally governed patient and HCP communication. Evidence from prior studies and our pilot testing of Loop itself does not show the overall volume of messages to be increased with the introduction of electronic communication [23,24]. However, there is no denying that Loop challenges the system to rethink the role of the patient and how HCPs communicate.

On the organizational and health system level, the accountability and payment incentive systems are often based on organizationally defined objectives. Coordination is not adequately compensated, posing an existential challenge to Loop. However, regardless of incentives, many HCPs spend a significant portion of their time chasing information and connecting with people to deliver safe, quality care. If Loop can save time in doing these tasks, the impact is obvious.

On a policy level, scaling Loop must consider complexities related to ownership of the system, privacy, data sharing, and regulatory approval. In a system organized in silos of funding, the method of payment for a cross-organizational tool like Loop is unclear. A broad coalition of partners is necessary for a collaborative project but difficult to translate into an effective governance and payment model.

### Conclusions

The development process of Loop shows the importance of grounding eHealth systems in clinical practice and patient experiences. Only through a robust research and UCD process is it possible to identify underlying issues and constraints. The core concept of open versus private communication evolved from the initial vision for open communication to partitioning the space to create an HCP-only view based on user perspectives and the preliminary pilot testing showing that open communication is predominantly being used. This demonstrates that the next phase of a clinical trial of Loop is a critical step in validation of the UCD. In the trial, we will evaluate whether the functionalities that emerged through our approach so far translate as intended in clinical practice and patient experience of Loop.

## Abbreviations

AYAC: adolescents and young adults with cancer
CMC: children with medical complexity
EHR: electronic health records
HCP: health care provider
ICT: information and communication technology
PHR: patient-held record
UCD: user-centered design
